# Optimising the decellularization of human elastic cartilage with trypsin for future use in ear reconstruction

**DOI:** 10.1038/s41598-018-20592-x

**Published:** 2018-02-15

**Authors:** Shafiq Rahman, Michelle Griffin, Anish Naik, Matthew Szarko, Peter E. M. Butler

**Affiliations:** 1 Division of Surgery and Interventional Science, University College London (UCL), London, United Kingdom; 2 Charles Wolfson Centre for Reconstructive Surgery, Royal Free Hospital, London, United Kingdom; 3 Plastic and Reconstructive Surgery Department, Royal Free Hospital, London, United Kingdom; 4 Anatomy Department, St George’s University, London, United Kingdom

## Abstract

Decellularized scaffolds can induce chondrogenic differentiation of stem cells. This study compares different methods to optimise the decellularization of auricular cartilage. The process consisted of an initial 12 hour dry freeze thaw which froze the cartilage specimens in an empty tube at −20 °C. Samples were allowed to thaw at room temperature followed by submersion in phosphate buffer solution in which they were frozen at −20 °C for a 12 hour period. They were then allowed to thaw at room temperature as before. Protocol A subsequently involved subjecting specimens to both deoxyribonuclease and sodium deoxycholate. Protocol B and C were adaptations of this using 0.25% trypsin (7 cycles) and a 0.5 molar solution of ethylenediaminetetraacetic acid (3 hours for each cycle) respectively as additional steps. Trypsin accelerated the decellularization process with a reduction in DNA content from 55.4 ng/μL (native) to 17.3 ng/μL (P-value < 0.05) after 14 days. Protocol B showed a faster reduction in DNA content when compared with protocol A. In comparison to protocol C after 14 days, trypsin also showed greater decellularization with a mean difference of 11.7 ng/μL (P-value < 0.05). Histological analysis with H&E and DAPI confirmed depletion of cells at 14 days with trypsin.

## Introduction

Malformations of the external ear are known to cause cosmetic^1,2^ and psychological disturbances^3^ with numerous etiologies ranging from burns, cancer, trauma and congenital defects. Physical aberrations of the ear have been reported as a significant factor in limiting social integration^4^ and plastic surgeons have used various operative techniques to correct them. Ear reconstruction, however, has been historically very challenging^5,6^ owing to the need to reconstruct the complex nature of the ear’s shape. Currently, surgeons use costal cartilage grafts to recreate the ear’s framework. However, this approach causes donor site morbidity^7^ as well as requiring extensive surgical expertise^8–10^. In addition, the histological make up of elastic cartilage differs to costal cartilage^11^ and this influences the mechanical properties with the auricular elastic cartilage possessing a higher degree of flexibility^12^. This is due to its extracellular matrix (ECM) possessing a high density of elastic fibres. Hyaline cartilage however has a rich amorphous gelatinous matrix with a high density of collagen type 2 fibres as well as chondroitin sulphate^11^. This discrepancy in mechanical properties does not make costal cartilage grafts ideal for ear reconstruction.

Since the advent of material science and biomaterials, other options have been experimented with. MedPor® ear scaffolds composed of porous polyethylene have been applied clinically, however, they have reported a high incidence of extrusion post implantation^13^. The use of different synthetic scaffolds for auricular repair has demonstrated some difficulties in mimicking the native ECM as well as cytotoxicity and degeneration obstacles^13^. An alternative option is the use of decellularized scaffolds, as they mimic the ECM. A native ECM offers a more biocompatible approach for auricular repair and decellularization of ear cartilage has so far successfully offered a scaffold, which is capable of inducing cartilage formation^14^. However, the understanding into decellularization protocols for human auricular cartilage is limited. In the study by Utomo *et al*.^15^, the number of cycles required to achieve optimum decellularization of ear cartilage is not specified, this makes it difficult to replicate by other research groups. Also their study design does not compare different decellularization protocols which limits the evaluation of their proposed method. Gong *et al*.^16^ have briefly referred to a protocol however they do not provide intricate detail of the experimental steps. In addition, their decellularized cartilage was of a porcine source.

The aim of this study was to identify a protocol that could optimize the decellularization of human auricular cartilage. The technique was based on the success of previous methods applied in the case of tracheal^17,18^ and ear decellularization^15^. Our methodology consisted of applying three different decellularization protocols to human ear cartilage consisting of both physical and chemical methods. Physical methods of decellularization such as freeze thaw can help disrupt cell membranes^19^ as well as induce apoptosis of chondrocytes^20^. However the effect on degradation of the extracellular matrix structure has been proven to be insignificant. Szarko *et al*. reported no differences in glycosaminoglycan and collagen content after freeze thaw^21^ as well as reporting no change in the mean complex stiffness level. The process however initiates decellularization by inducing necrosis of cells whilst maintaining the three dimensional matrix structure. This reduces the duration of exposure to chemical methods of decellularization^19^. Chemical methods whilst effective can impair the mechanical properties of the matrix as Elder *et al*. noted decreased matrix strength when the exposure time of cartilage to SDS was increased from 2 hours to 8 hours^22^. Therefore a combination with physical decellularization techniques can help reduce the exposure time of cartilage to chemical methods. Freeze thaw action has been reported to accelerate the decellularization process in various tissues with lower nuclear content detected when combined with chemical techniques^23^. This study is the first to compare multiple decellularization protocols for human auricular cartilage and in doing so has identified a pathway for accelerating the process whilst preserving the structure of the native matrix.

## Materials and Methods

### Ethics

Ethical approval for use of cadaveric cartilage was obtained in accordance with the human tissue act 2004. Local ethical approval for medical education and research was obtained from St Georges University, London.

### Cartilage harvest

**Figure 1:**
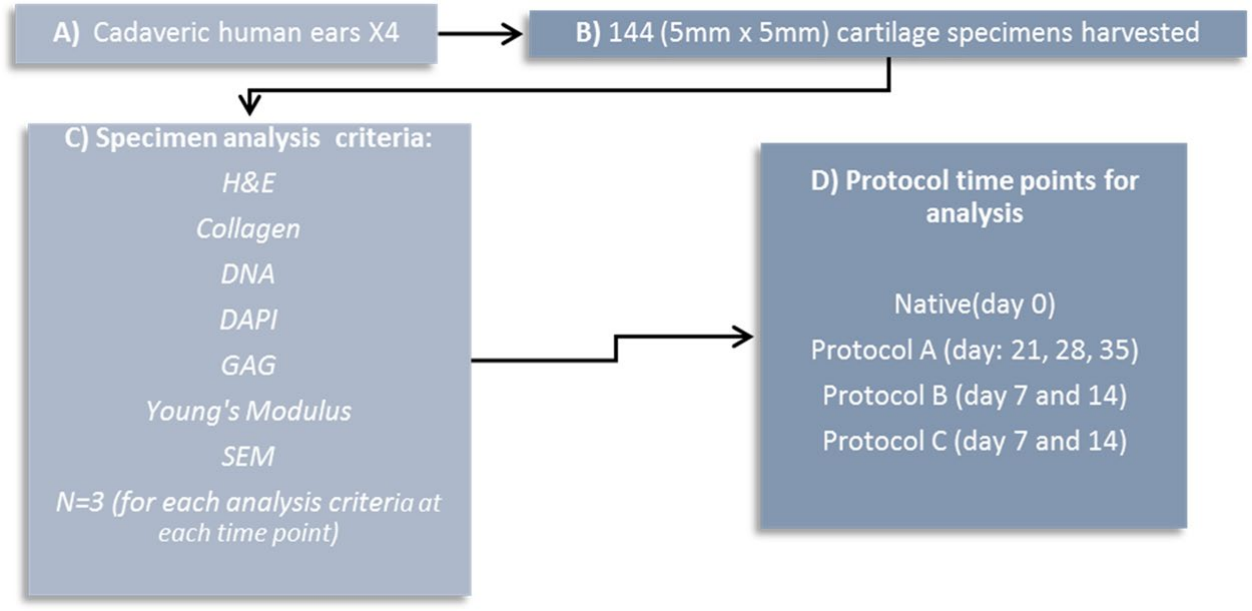
Summary of steps for cartilage harvest.

Procurement of auricular elastic cartilage was conducted by careful dissection of cadaveric human ears removing the skin and underlying perichondrium. This was subsequently followed by harvesting 5 mm × 5 mm sections from the elastic ear cartilage. Specimens were then split up into assessment for GAG (glycosaminoglycans), DNA, collagen and for histological analysis with H&E, DAPI, Masson’s Trichrome, Scanning Electron Microscopy (SEM) as well as assessing for mechanical properties (n = 3). For native tissue a total of 21 samples were used. Figure 1 provides a summary of the steps involved and details of analysis time points as well as assessment criteria.

### Decellularization process

#### Protocol A

**Figure 2:**
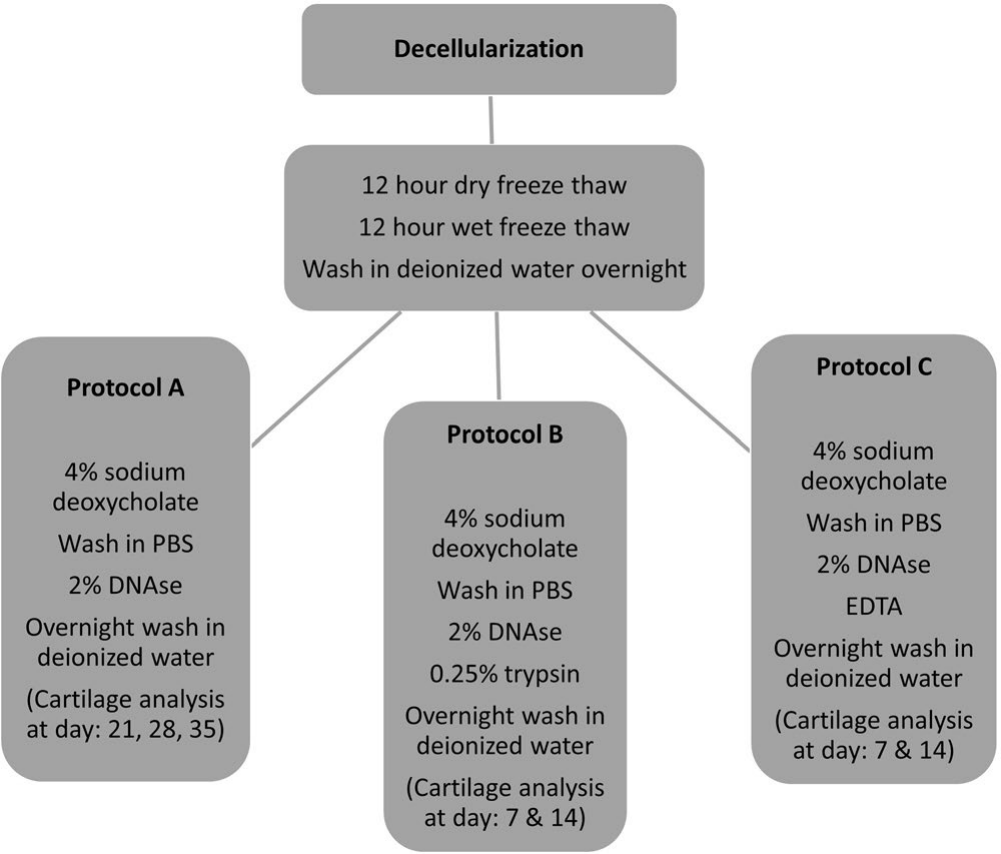
Summary of steps for protocol A, B and C.

The protocol started with an initial dry 12 hour freeze thaw of the specimens followed by thawing at room temperature. This was followed by another 12 hour wet freeze thaw cycle in phosphate buffer solution (PBS, Sigma-Aldrich, USA) at −20 °C. The addition of a wet freeze thaw cycle in PBS allowed for cellular remnants to be washed away from the scaffold after thawing at room temperature. In addition it exerted greater mechanical pressure on the cartilage in physically decellularizing it. The cartilage was subsequently washed in deionized water overnight under agitation at room temperature. The next step involved transferring the tissue specimens to 4% sodium deoxycholate solution (Sigma-Aldrich, USA) under agitation at room temperature for four hours. This was subsequently followed by a wash in PBS solution for 30 minutes under agitation at room temperature. Next, the tissue specimens were submersed in 2% deoxyribonuclease/DNase (Sigma-Aldrich, USA) for 3 hours. This was followed by an overnight wash in deionized water with agitation at room temperature. The process was repeated for subsequent cycles however the initial freeze thaw steps were only performed once at the beginning of the protocol (Fig. 2).

#### Protocol B

Protocol B was an adaptation of protocol A and involved the same steps however specimens were submersed in 0.25% trypsin(Sigma-Aldrich, USA) for three hours after being subject to treatment with 2% DNAse (Sigma-Aldrich, USA). This was followed by an overnight wash in deionized water as before. Trypsin was used only for 7 cycles so as to avoid damaging the ECM.

#### Protocol C

Protocol C was again a modification of protocol A with similar steps however it involved subjecting the specimens to 0.5 M ethylenediaminetetraacetic acid (EDTA, Sigma-Aldrich, USA) for 3 hours after treatment with 2% DNAse (Sigma-Aldrich, USA).

#### H&E

Following fixation in 4% paraformaldehyde (PFA), the specimens were paraffin embedded. They were then subjected to dehyrdration and rehydration cycles. Specimens were subsequently stained in Hematoxylin for 10 minutes. They were then briefly washed in running tap water for 3 seconds after which they were differentiated in acid alcohol for 6 seconds. This was a 1% concentration solution consisting of hydrochloric acid and 70% ethanol. Specimen were run through tap water until becoming blue in colour. Staining was conducted in 1% eosin for 5 minutes. Slides were analysed with the use of light microscopy (EVOS, XL Core) (n = 3).

#### Masson’s Trichrome

Following fixation in 4% PFA, the specimens were paraffin embedded and were then subjected to dehydration and rehydration cycles. Nuclei were subsequently stained with celestine blue-haemalum sequence and rinsed in distilled water. Subsequent staining was conducted with Hematoxylin for 5 minutes. Staining was then conducted in ponceau-acid fuchsin solution for 5 minutes. Specimens were later differentiated in 1% aqueous phosphotunstic acid for 10 minutes. Counterstain in light green was performed. Specimens were finally dehydrated and mounted on to slides and examined with light microscopy (EVOS, XL, Core) (n = 3).

#### DAPI Analysis

Preparation of slides for 4,6-diamidino-2-phenylindole (DAPI) staining involved incubation of the slides at 60 °C to remove wax in which they were initially fixated within. This was for 30 minutes. They were then submersed three times with stock solution of xylene for 5 minutes each time. After this step, they were taken through absolute IMS for 5 minutes, followed by 90% IMS for 5 minutes and then 70% IMS. Finally, one drop of DAPI stain was applied. Three slides were prepared with DAPI for each time point from the different protocols and assessed under fluorescent microscopy (Invitrogen FL Cell Imaging System) (n = 3).

#### DNA Content

DNA measurement was conducted with a DNeasy quantification kit supplied by Qiagen and performed according to manufacturer conditions. A proteinase K–Buffer ATL working solution was prepared which contained 20 μl proteinase K stock solution and 180 μl Buffer ATL per sample. Mixing by vortex and centrifugation at 3000 rpm for 10 minutes was performed and the samples were then incubated overnight at 56 °C to ensure lysis of the tissue. Following this, 410 μl of premixed Buffer AL–ethanol was added to each sample. After vigorous vortex for 15 seconds, samples were then centrifuged again at 3000 rpm for 10 minutes to allow homogenous distribution of the lysate. The supernatant was then removed from each sample and centrifuged again at 6000 rpm for ten minutes. Then 500 μl of buffer AW1 was added to each sample and re-centrifuged for 5 minutes at 6000 rpm. Next, 500 μl of the Buffer AW2 was added to each one of the samples. These were centrifuged for 15 minutes at 6000 rpm. A nanodrop spectrophotometer (Thermo Scientific NanoDrop Lite Spectrophotometer) was used to measure the DNA concentration at a 260 nm wavelength (n = 3).

#### GAG Assay

Glycosaminoglycan (GAG) content was measured using the blyscan sulfated glycosaminoglycan assay kit (Biocolour, UK) according to manufacturer conditions.

In brief, the tissue samples being placed within eppendorf tubes after which the volume was made up to 100 μl with distilled water. Then 1 ml of Blyscan dye reagent was added to each specimen. Glycosaminoglycan standards were then prepared by employing aliquots with the following concentration of GAG: 1.0, 2.0, 3.0, 4.0 & 5.0 μg. The specimens were subsequently placed on a mechanical agitator at room temperature for 30 minutes to allow binding of the blyscan dye to the sulphated glycosaminoglycans. The next step involved centrifuging the specimens at 10000 rpm for 10 minutes. The specimens were then inverted and drained removing the unbound dye solution. The next step involved releasing of the bound dye, which, was achieved by adding 1 ml of dissociation reagent to each one of the cartilage samples. The specimens were then mixed by way of a vortex. The contents were transferred to a well plate which was clearly labelled. This was inserted within a photospectrometer to calculate absorption ratios of the samples. The absorption ratios for the standards were plotted on a graph and these were used to calculate the content of GAG (n = 3).

#### Collagen assay

Collagen content was measured using the Quickzyme Biosciences (UK) kit and performed according to manufacturer conditions. In brief the cartilage specimens were inserted within eppendorf micro-centrifugation tubes. To each sample 6 M HCl was then added and made up to a volume of 100 μl. The specimens were then incubated at a temperature of 95 °C for 20 hours in an oven to commence hydrolysis. They were then allowed to cool at room temperature. Next the samples were centrifuged for 10 minutes at 13000 rpm. The supernatant was then transferred from each tube to fresh ones whilst taking care not to pipette the black particles which represented fat degradation products. These can interfere with the absorption of light. 100 μl of water was added to each one of the samples so as to dilute the hydrolysate. Then 35 μl of the new mixture was used for analysis with a spectrophotometer (Thermo Scientific Fluoroskan Ascent FL Microplate Fluorometer and Luminometer). It contained a Quartz-halogen pump, filters as well as a photomultiplier tube. It had an emission wavelength range of 360 nm to 670 nm. The fluorometric sensitivity was 2 fmol fluorescein/well in a black 96 well plate (n = 3).

### Scanning electron microscopy

Following fixation in 4% PFA for 48 hours the specimens were washed in deionized water for 10 mins. The specimens were then dehydrated in ascending ethanol washes (50%, 70, 90%, 100%) for 10 mins each. Following gold coating, the specimens were placed on carbon coated alumminin stubs before being imaged (FEI Quanta 200 F, n = 3).

### Mechanical properties

Cartilage specimens were tested using indentation compression using a Mach-1 material testing machine (Biomomentum, Canada). Each specimen was loaded to 300 g at 1 mm/sec via a 1 kg load cell. The Young’s modulus was reported for native tissue as well as the end points for protocol A at day 35, protocol B and C at day 14 with *N* = 3 at each time point. In addition, the percentage weight reduction in comparison to native for the different protocols was recorded.

### Statistical analysis

All statistical analysis was conducted with the aid of SPPSS version 24. One way ANOVA as well as the standard T-test were applied for comparing the mean content of DNA, GAG and collagen across our different protocols including their separate time points. Further analysis was conducted through post-HOC bonferoni assessment.

## Results

### Histological analysis

**Figure 3:**
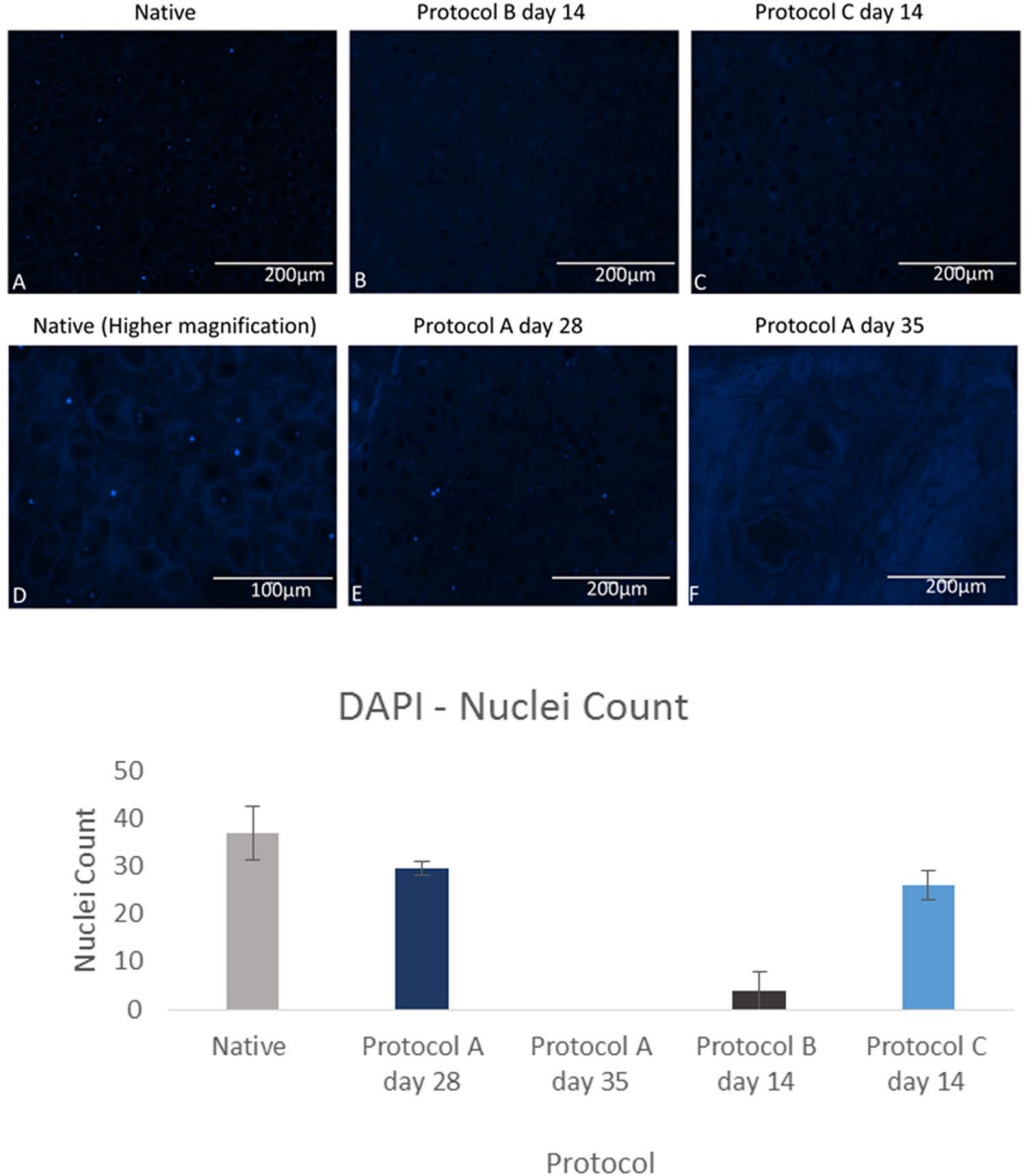
DAPI (4′,6′-diamidino-2-phenylindole) analysis of cartilage specimens assessing for decellularization with quantification of nuclei count per slide (n = 3) for different protocols using imageJ. Error bars for standard deviation.

This was performed with DAPI as well as H&E which are shown in Figs 2 and 3 respectively. Collagen was also assessed with masons trichrome. Cell quantification was conducted using imageJ software with intact nuclei in each image being counted using the cell counter plugin for the DAPI and H&E slides.

**Figure 4:**
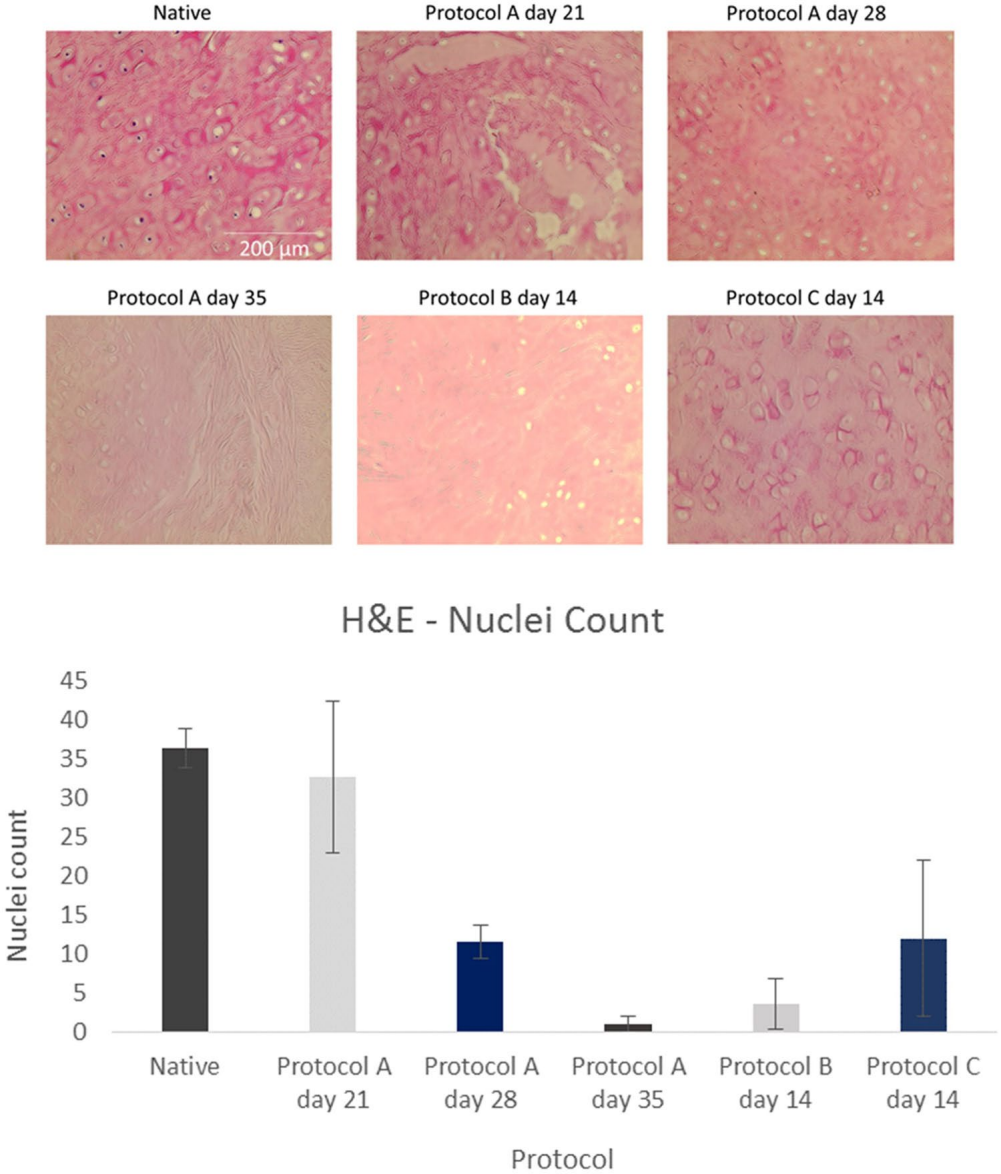
H&E stained cartilage sections demonstrating decellularization of native tissue with quantification of nuclei count per slide (n = 3) for different protocols using imageJ. Error bars for standard deviation.

Assessing the nuclear count with DAPI showed that both protocol B at day 14 and protocol A at day 35 had a significantly lower cell number in comparison to native (P-value < 0.05). These results have been confirmed with H&E staining (Fig. 4) which too has shown a significant reduction in nuclear count for both protocol B at day 14 and protocol A at day 35 (P-value < 0.05) relative to native samples.

Purple stained nuclei evident within native cartilage of H&E stained specimens (Fig. 4). Progressive loss of nuclei with increased exposure to detergent/enzymatic decellularization in protocol A with no nuclei visible after 35 days. Protocol B demonstrates that after 14 days of decellularization using trypsin for the first 7 cycles, adequate decellularization can be achieved. Figure 4 displays absence of nuclei after 14 days. In the absence of trypsin as in protocol A, 35 days were required to achieve a similar histological picture of decellularization.

Protocol C with EDTA demonstrates that purple stained nuclei are still evident after 14 days of decellularization. The trypsin protocol however has shown to be quicker in removing cells with fewer cellular remnants visible after 14 days. This has been confirmed with cell quantification with protocol B at day 14 having a 0.70 fold lower mean cell count compared to protocol C at the same time point.

### Collagen

**Figure 5:**
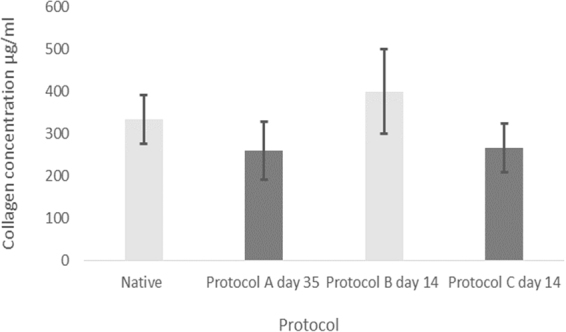
Mean collagen concentration for native samples and the different protocols with error bars for standard deviation. ANOVA analysis (P value: 0.076) indicating no significant difference between the respective study groups.

The collagen content was found to either remain relatively the same as native or to increase in concentration for all protocols (Fig. 5).

**Figure 6:**
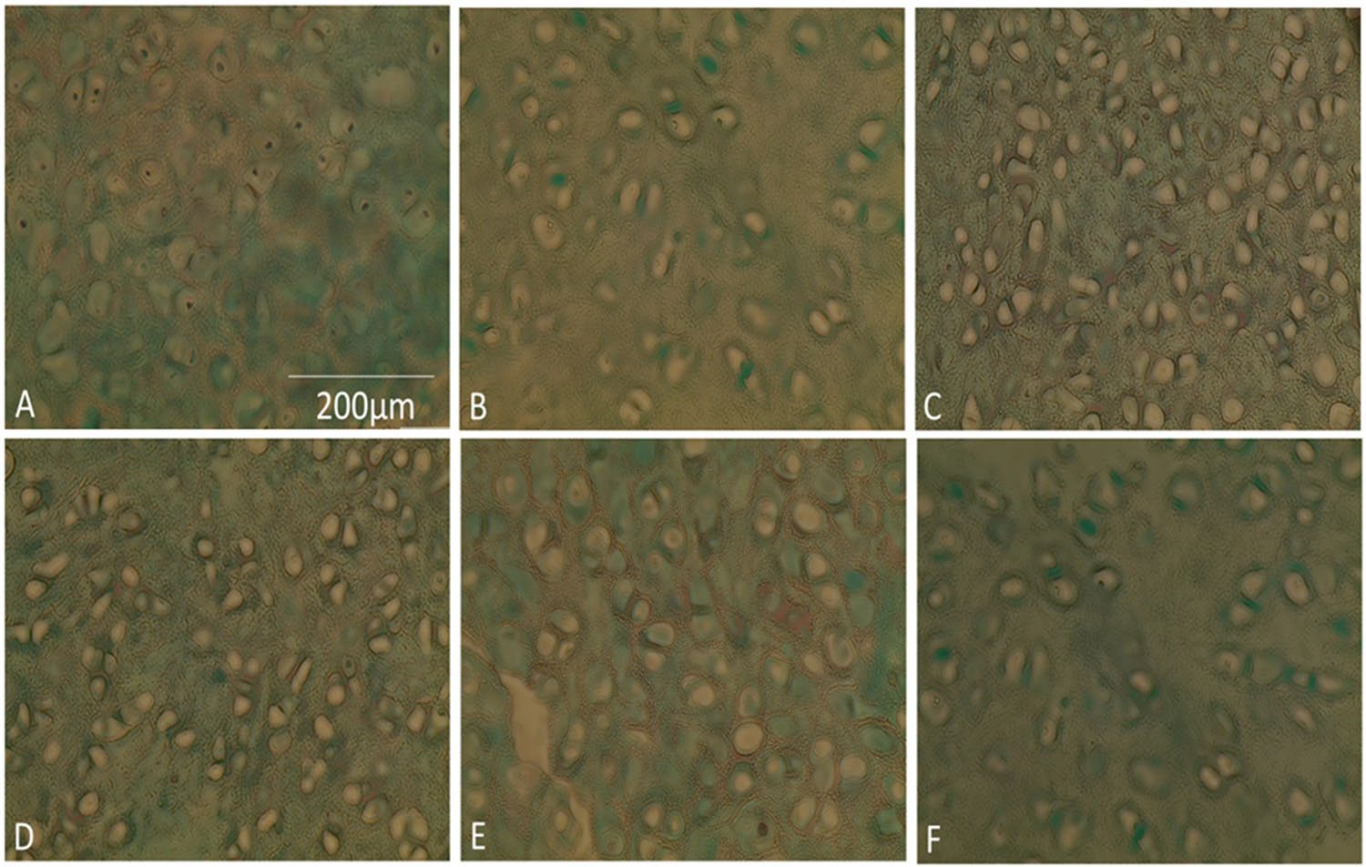
Masson’s trichrome stain of elastic cartilage. Same density distribution of collagen fibres between lacunae amongst different protocol groups. (**A**) Native. (**B**) Protocol A, day 28. (**C**) Protocol B, day 7. (**D**) Protocol B, day 14. (**E**) Protocol C, day 7. (**F**) Protocol C, day 14.

For protocol A at day 35 there was a 0.22 fold reduction in collagen after decellularization compared to native which was not significant (P-value: 0.23). The same was the case for protocol C at day 14 where there was a comparable reduction of only 0.2 fold (P-value: 0.23). In protocol B at day 14 there was an increase in collagen content compared to native of 20% from 333 μg/ml to 400 μg/ml however this was insignificant (P-value: 0.37). There was no change in collagen content between day 7 and 14 of protocol B with mean values of 400 μg/ml at both time points. For protocol C the average collagen content decreased from 300 μg/ml at day 7 to 267 μg/ml at day 14 which marked a 0.11 fold reduction however this was not considered significant. Comparison of the collagen content for native cartilage, day 35 of protocol A, day 14 of protocol B and C showed no significant difference at these time points (P-value: 0.076). This has been reflected by histology below where there was a similar density of collagen stained with masons trichrome amongst the different protocols (Fig. 6).

**Figure 7:**
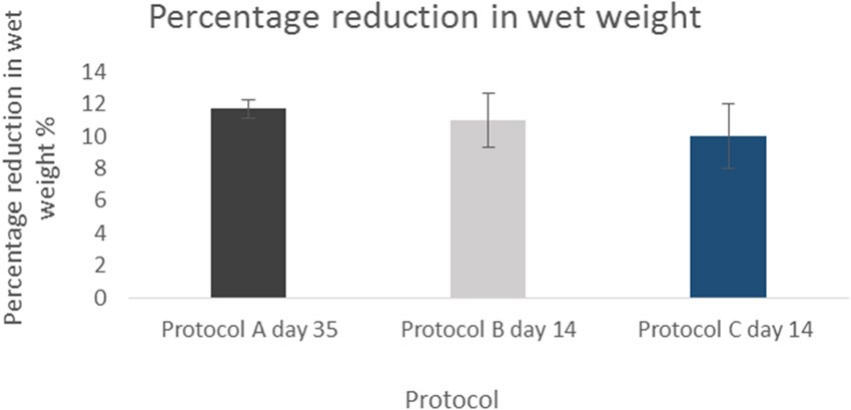
Mean percentage reduction in wet weight compared to native *(N* = *3)*. Error bars for SD.

The results for collagen content follows the effect of normalization^15^ with depletion of GAG allowing for collagen to redistribute within the structure of ECM, therefore appearing to measure at an almost similar concentration with no statistical difference to native. This is due to a decrease in the wet weight of the cartilage specimens. The collagen level measures in association to a reduced wet weight post decellularization therefore appearing at a higher concentration. The wet weight decreased consistently for all protocols at their end points. The percentage decrease relative to native tissue is demonstrated in Fig. 7.

### GAG

The results depict that GAG content was reduced the most when samples were tested on day 35 of protocol A as it was undetectable (Table 1). Protocol B at day 14 experienced a 0.88 fold reduction and protocol C at day 14 underwent a comparable decrease of 0.94 fold compared to naïve (Table 1). Assessment of protocol A at day 28 as well as protocol B and C at days 14 showed a significant difference at these time points (P-value < 0.05, one away ANOVA). Post-HOC bonferoni assessment of protocol B (day 14) and protocol C (day 14) was insignificant (P-value: 0.072) but protocol B (day 14) showed a significantly higher GAG level compared to protocol A, day 28 (P-value: 0.022). Assessment of protocol A (day 28) and day 14 of protocol C also demonstrated a significantly higher GAG level (P-value < 0.05) in the latter group. All time points underwent a reduction in GAG content when compared to native (P-values < 0.05).

**Table 1:** Mean concentration of GAG for different time points.

Protocol	Mean GAG concentration (n = 3)
Native	3.3 μg
Protocol A day 21	0.1 μg
Protocol A day 28	0.1 μg
Protocol A day 35	<0.1 (undetectable)
Protocol B day 7	0.5 μg
Protocol B day 14	0.4 μg
Protocol C day 7	0.8 μg
Protocol C day14	0.2 μg

Normalising the GAG content to percentage reduction in wet weight shows that protocol A at day 35 underwent the greatest depletion in GAG level which corresponded with the highest reduction in the wet weight fraction (Fig. 7). This effect was also seen in protocol B at day 14 which demonstrated the second highest reduction in GAG and wet weight fraction.

### DNA

Protocol A at day 35 demonstrated a reduction in the DNA content when compared to native (P-value: 0.0026). This was also the case at day 28 (P-value < 0.05). Day 21 however did not produce a significant reduction in the content of DNA compared to native (P-value: 0.074).

Comparing the DNA content at the end of 14 days of protocol B with native, a significant reduction was seen (P-value < 0.05). Similar findings were deduced for protocol C at day 14 (P-value < 0.05).

**Figure 8:**
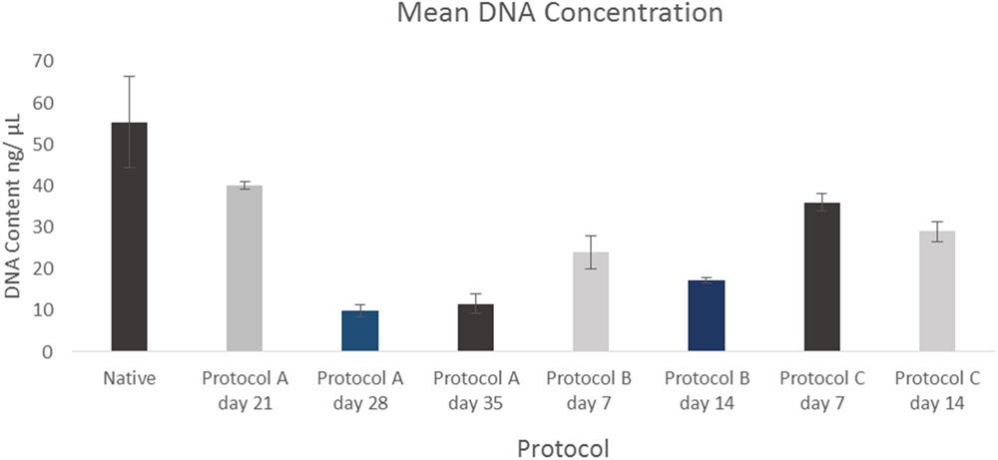
Mean DNA content for different protocols (*N* = 3). Error bars for SD.

There was a significant difference between day 35 of protocol A as well as day 14 of both protocol B and C (P-value < 0.05). Significant differences also existed between protocol A at day 35 and protocol C at day 14 (P-value < 0.05) as well as between protocol B, day 14 and protocol C, day 14 (P values: <0.05). However there was believed to be no difference between protocol A day 35 as well as protocol B day 14 (P-value: 0.23). Results for DNA are demonstrated in Fig. 8.

Normalising DNA content to the percentage reduction in the wet weight fraction there was a consistent decrease at the end points of the three protocols (Fig. 7) correlating with a decrease in DNA content. Protocol A at day 35 underwent the highest reduction (0.79 fold) in DNA content compared to native and equally showed the greatest percentage reduction in wet weight fraction. The same trend was observed in in the case of protocol B and C at 14 days with the former showing a greater decrease in DNA content and reduction of the wet weight fraction.

### Scanning electron microscopy (SEM)

**Figure 9:**
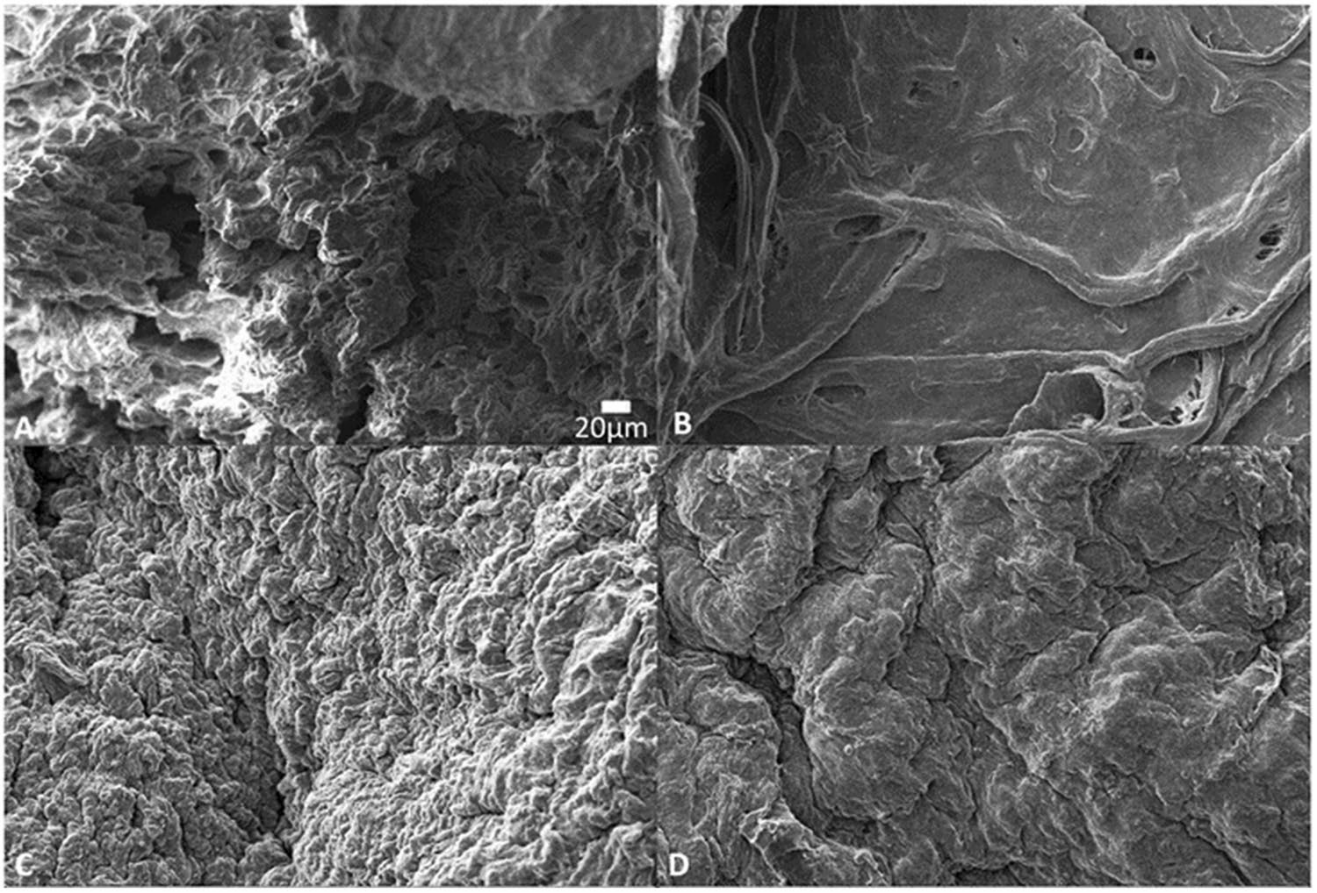
Scanning electron microscopy of cartilage specimens (high magnification). (**A**) Native. (**B**) Protocol A, day 35. (**C**) Protocol B, day 14. (**D**) Protocol C, day 14.

SEM was used to assess the cartilage structure. Results of SEM (Fig. 9) showed obliteration of the matrix structure in protocol A at day 35 in comparison to native. Protocol B and C however showed preservation with a similar morphology to that of native.

The innate complex surface structure of the ECM is comparable in appearance between native cartilage as well as protocols B and C at day 14 under scanning electron microscopy.

### Mechanical properties

**Figure 10:**
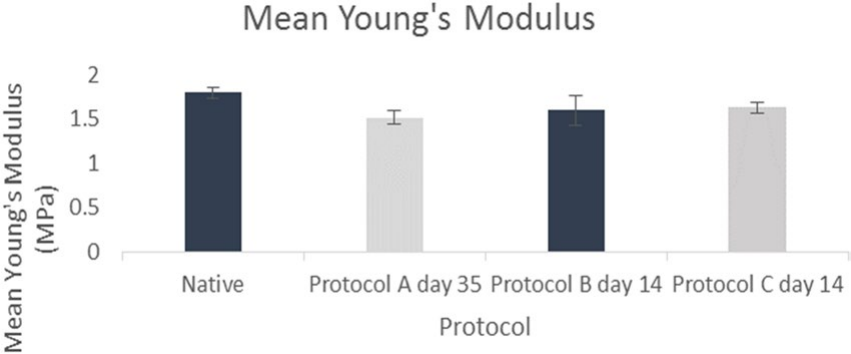
Mean Young’s modulus (MPa) for native samples, protocol A, B and C (*N* = *3*). Error bars for standard deviation.

The Young’s modulus decreased significantly post decellularization in comparison to native samples for protocol A at day 35 (P < 0.05). For protocol B at day 14 however there was no significant difference on comparison to native (P value: 0.1270). This was also the case for protocol C at day 14 (Fig. 10).

## Discussion

Literature on decellularization of human auricular cartilage is currently in-sufficient. Utomo *et al*.^15^ have provided a protocol however it didn’t specify the number of decellularization cycles required to produce an optimized scaffold. Similarly in the study by Gong *et al*.^16^, they have not clarified details of experimental steps. In addition the cartilage is of a porcine source only. This study is the first to compare multiple protocols for decellularization of human auricular cartilage with the detergent/enzymatic method proposed in protocol B having shown to have been the most effective. The steps for protocols A to C were chosen based on the success of methods from previous study groups. The process of freeze thaw has shown to accelerate decellularization^23^ when applied to different tissues and this can minimize the duration of exposure to chemical forms. Adding a wet freeze thaw cycle in PBS allowed for cellular remnants to be washed away from the scaffold after thawing at room temperature as well as exerting greater mechanical pressure on the cartilage in physically decellularizing it. Detergent/enzymatic methods can damage the structure of the ECM if prolonged^22^ and so physical techniques can shorten the exposure time. The use of EDTA in protocol C was adapted from Utomo *et al*.^15^ who had used it successfully in their decelullarization process. In addition the use of DNAse as well as SDC amongst the different protocols has been reported to effectively decellularize cartilage in the literature^17^. Trypsin was used in protocol B as it has shown to accelerate decellularization in the case of hyaline cartilage of the trachea as reported by Gomez *et al*.^18^.

Data in this study has shown that trypsin can certainly be effective. Histological analysis with DAPI stain has demonstrated that cells were successfully removed by day 14 with a significant reduction in comparison to native. Findings were supported by the H&E results which demonstrated a similar pattern to the DAPI staining. This was statistically proven with DNA showing a significant reduction after 14 cycles to native (P < 0.05). Staining with Masson’s Trichrome showed that collagen was still present within the extra cellular matrix which is important since it’s vital to maintain the biomechanical properties as well as acting as a biochemical cue when it comes to directing cell behaviour^19^. In addition the mean Young’s Modulus was maintained post decellularization at the end of protocol B compared to native.

 SEM showed that the three dimensional hierrarchial arrangement of the matrix was maintained at day 14 for protocol B. Protocol A was not ideal since it not only necessitated 35 days to decellularize the cartilage but in doing so it obliterated the ECM’s three dimensional (3D) structure. Surface characterization of cartilage post decellularization with SEM has also been reported by Utomo^15^ as well as Gomez^18^ and is an effective method of ECM analysis to asses for gross changes in the scaffold’s structure.

Glycosaminoglycans constitute an important part of the ECM structure for providing biochemical cues to guide cell behaviour^24^. Numerous studies have been conducted where their presence has been beneficial for directing differentiation of cells. Murphy *et al*.^24^ have emphasized the importance of GAG in increasing differentiation of mesenchymal stem cells towards a chondrogenic lineage. The role of intrinsic ECM properties including GAG content influencing cell behaviour has been demonstrated by Reilly *et al*. also^25^. Whilst it’s important to retain GAG for influencing stem cell activity, a reduction in content may also have a benefit as it reduces the density of the ECM and increases pore size^26^. This would allow for greater cellular infiltration if recellularization is attempted. Therefore the ideal scaffold for cartilage should have a balance of reduced GAG whilst not completely depleting its level so that cell differentiation can be guided. This has certainly been reflected by the scaffold produced in the trypsin protocol within this study where GAG has been reduced but not completely lost. Utomo *et al*.^15^ have reported an almost similar outcome for decellularization of ear cartilage with a 75.3% reduction compared to an 88% for the trypsin protocol at day 14 in this study.

Whilst the depletion of DNA to a significant amount was desirable for the decellularization pathway in protocol A, a 35 day cycle however lead to significant morphological changes with denaturation of the 3D ECM structure on SEM when compared with native. Protocol C with EDTA did not produce the same level of reduction in DNA as did protocol B with trypsin after 14 cycles of chemical decellularization. Protocol B however, was able to reduce it in a short time whilst preserving the three dimensional structure of the cartilage.

Results demonstrated that the average collagen content either increased or stayed the same. This phenomenon of normalization has previously been reported within the literature^15^. It occurs as a result of a decrease in the GAG content after decellularization. This induces a compensatory redistribution of the collagen content and an increase to the wet weight contribution of cartilage. Similar results have been obtained in other studies^15^. Collagen is vital for provision of biomechanical strength to the scaffold as well as guiding chondrogenic differentiation^27^. Therefore it is important that it is retained within the native ECM. Assessment with Masson’s Trichrome  showed that collagen was retained post decellularization and this is comparable to the study by Utomo^15^ in which immunohistochemical staining has also shown the retention of collagen fibres.

This study has so far optimized the decellularization process for human auricular cartilage. This technique can be incorporated further with biofabrication strategies in developing hybrid scaffolds for auricular reconstruction. Biofabrication through the use of 3D printing has been able to recreate the complex shape of human ears^28^ and enabled targeted cell seeding as well as growth factor delivery^29^. It has allowed for the use of hydrogels also termed “bio-inks” to be coated on to three dimensional ear scaffolds providing an aqueous environment in which chondrogenic differentiation of stem cells can occur^12^. Bio-inks can also be constituted by soluble forms of decellularized extra cellular matrices which have shown to be more biocompatible options^30^. By optimizing the production of a decellularized ECM, this can be combined with bioprinting strategies to improve the overall process of auricular regeneration.

This study has identified a successful process for producing an acellular elastic cartilage ECM for ear reconstruction. Further work will involve cell seeding on to the scaffold to understand biocompatibility.

## Conclusion

Human ear cartilage can be successfully decellularized in the production of cartilage scaffolds. Using trypsin, the decellularization process produces scaffolds within a 14 day period that have optimal structural and biomechanical properties which mimic the native ECM. The use of decellularized scaffolds for elastic cartilage regeneration looks promising for auricular reconstruction.
